# Advances in Immunotherapeutics in Pancreatic Ductal Adenocarcinoma

**DOI:** 10.3390/cancers15174265

**Published:** 2023-08-25

**Authors:** Tarak Chouari, Francesca Soraya La Costa, Nabeel Merali, Maria-Danae Jessel, Shivan Sivakumar, Nicola Annels, Adam E. Frampton

**Affiliations:** 1 Hepato-Pancreato-Biliary Department, Royal Surrey NHS Foundation Trust, Guildford GU2 7XX, UK; t.chouari@nhs.net (T.C.); f.lacosta@nhs.net (F.S.L.C.); n.merali@nhs.net (N.M.); 2 Section of Oncology, Department of Clinical and Experimental Medicine, Faculty of Health and Medical Sciences, University of Surrey, Guildford GU2 7WG, UK; m.jessel@surrey.ac.uk (M.-D.J.); n.annels@surrey.ac.uk (N.A.); 3 The Minimal Access Therapy Training Unit, University of Surrey, Guildford GU2 7WG, UK; 4 Oncology Department and Institute of Immunology and Immunotherapy, Birmingham Medical School, University of Birmingham, Birmingham B15 2TT, UK; s.sivakumar@bham.ac.uk

**Keywords:** pancreatic ductal adenocarcinoma, immunotherapy, pancreas, vaccination therapies, oncolytic viral therapy

## Abstract

**Simple Summary:**

Pancreatic ductal adenocarcinoma (PDAC) is the most common type of pancreatic cancer, responsible for the majority of cases and ranking seventh as a leading cause of cancer-related deaths. It is difficult to treat because is it often detected at advanced stages, there are no effective screening tests available, and patients can develop resistance to standard treatments like chemotherapy and radiation. Recent interest has involved immunotherapy, which stimulate the immune system to recognise and attack cancer cells. Therefore, our paper aims to summarise the findings of studies investigating immunotherapies in PDAC and we discuss the limitations of such therapies and avenues of future research. In a bid to address the outcomes associated with the disease.

**Abstract:**

Pancreatic ductal adenocarcinoma (PDAC) accounts for up to 95% of all pancreatic cancer cases and is the seventh-leading cause of cancer death. Poor prognosis is a result of late presentation, a lack of screening tests and the fact some patients develop resistance to chemotherapy and radiotherapy. Novel therapies like immunotherapeutics have been of recent interest in pancreatic cancer. However, this field remains in its infancy with much to unravel. Immunotherapy and other targeted therapies have yet to yield significant progress in treating PDAC, primarily due to our limited understanding of the disease immune mechanisms and its intricate interactions with the tumour microenvironment (TME). In this review we provide an overview of current novel immunotherapies which have been studied in the field of pancreatic cancer. We discuss their mechanisms, evidence available in pancreatic cancer as well as the limitations of such therapies. We showcase the potential role of combining novel therapies in PDAC, postulate their potential clinical implications and the hurdles associated with their use in PDAC. Therapies discussed with include programmed death checkpoint inhibitors, Cytotoxic T-lymphocyte-associated protein 4, Chimeric Antigen Receptor-T cell therapy, oncolytic viral therapy and vaccine therapies including KRAS vaccines, Telomerase vaccines, Gastrin Vaccines, Survivin-targeting vaccines, Heat-shock protein (HSP) peptide complex-based vaccines, MUC-1 targeting vaccines, Listeria based vaccines and Dendritic cell-based vaccines.

## 1. Introduction

Pancreatic ductal adenocarcinoma (PDAC) accounts for up to 95% of all pancreatic cancer cases and is the fifth-leading cause of cancer death in the United Kingdom (UK) [1,2]. Over the last decade, mortality rates have continued to rise [2]. Such dismal outcomes are a consequence of the late stage at which PDAC is initially diagnosed coupled with a lack of screening tests for early detection. Furthermore, poor prognosis is further aggravated by resistance to chemotherapy and high recurrence rate [1,2,3].

Currently, surgery provides the only curative option. However, less than 20% of PDAC patients are eligible for surgery, as patients often have synchronous metastatic disease at initial presentation and/or the tumour is deemed borderline resectable or unresectable [2,4]. Patients with resectable PDAC have less than 5–10% chance of surviving up to 5 years [1]. Despite advances in surgical techniques, peri-operative care and chemotherapy/radiotherapy, there has been little progress in survival outcomes [1,2,3,5,6,7].

Immunotherapy and vaccination therapy work by identifying and targeting non-self-antigens that present on the tumour surfaces using the body’s own immune mechanism. They have been of recent interest in the management of PDAC [7,8,9,10,11,12,13,14,15,16,17,18]. It has been suggested that immunotherapy may have fewer side effects and offer less tumour-resistance compared to conventional therapies [7]. Immunotherapeutic agents may prove promising both to the patient, and the healthcare system. However, this field of study in PDAC remains in its infancy and a greater depth of understanding of novel therapies for pancreatic cancer is urgently required.

In this review, we provide an overview of current studies examining the role for immunotherapy in patients with PDAC. We will summarize the relevance of the proposed mechanisms of such cancer therapeutics, the evidence underpinning their efficacy (or lack of) and address the role novel therapies may play in the future management of PDAC. Furthermore, in this review, we ask ourselves, what are the hurdles and clinical implications associated with their clinical application and how can we overcome them?

Therapies discussed with include programmed death checkpoint inhibitors, Cytotoxic T-lymphocyte-associated protein 4, Chimeric Antigen Receptor-T cell therapy, oncolytic viral therapy and vaccine therapies including KRAS vaccines, Telomerase vaccines, Gastrin Vaccines, Survivin-targeting vaccines, Heat-shock protein (HSP) peptide complex-based vaccines, MUC-1 targeting vaccines, Listeria based vaccines and Dendritic cell-based vaccines.

## 2. Immunotherapeutics

### 2.1. Programmed Death Checkpoint Inhibitors

Programmed death checkpoint inhibitors block the PD-L1 and PD-L2 (programme death protein ligands 1 & 2), which are usually associated with poor prognosis [11]. These ligands are upregulated in tumour cells and cause evasion of the body’s normal immunological response by inhibiting CD8^+^ T-cells proliferation in the tumour microenvironment (TME). With this evasion, the tumour may progress. PD-1/PD-L1 inhibitors activate and lead to proliferation of the T-cells which in turn attack the tumour directly [19,20].

As a monotherapy treatment, PD-1/PD-L1 inhibitors have shown promising results for certain malignancies [11]. The KEYNOTE-028 clinical non-randomised, multicentre, phase 1b single-arm trial (ClinicalTrials.gov identifier: NCT02054806) treated 475 patients, with incurable solid tumours unsuitable for or whom failed conventional therapies, with Pembrolizumab (PD-1 inhibitor) 10 mg/kg every 2 weeks for 2 years [21]. In this trial, the median (95% CI) progression free survival was 1.7 months (1.5 to 2.9 months). Those who survived longer had more PD-L1 tumour receptors on their surface, suggesting PD-L1 expression level may influence the response to PD-L1 inhibitors and thus improve survival. There may be potential for PD-L1 expression or a surrogate for its expression in identifying patients who would benefit from such therapies. However, some PD-L1 positive patients showed poor response to PD-L1 inhibitors whilst some PD-L1 negative patients respond well to PD-L1 inhibitors. Suggesting other ligands of PD-1 like PD-L2 may be relevant to the efficacy of PD-1 axis immunotherapy in several cancers investigated in the KEYNOTE-028 trial. There is some work which which corroborates that PD-L2 influences the anti-PD1 axis, specifically in PDAC [22,23,24].

The greatest efficacy reported for PD-1inhibitor monotherapy was reported in a phase I trial investigated the effects of Pembrolizumab in an advanced PDAC patient, who had disease-free progression for 20 weeks after anti-PD-1 treatment [25]. Furthermore, a phase I/II clinical trial (NCT03829501) assessing the efficacy of atezolizumab (PD-L1 inhibitor) in advanced PDAC, as well as other tumours refractory to first-line treatment who are ineligible for other available treatments, is currently ongoing.

**Figure 1:**
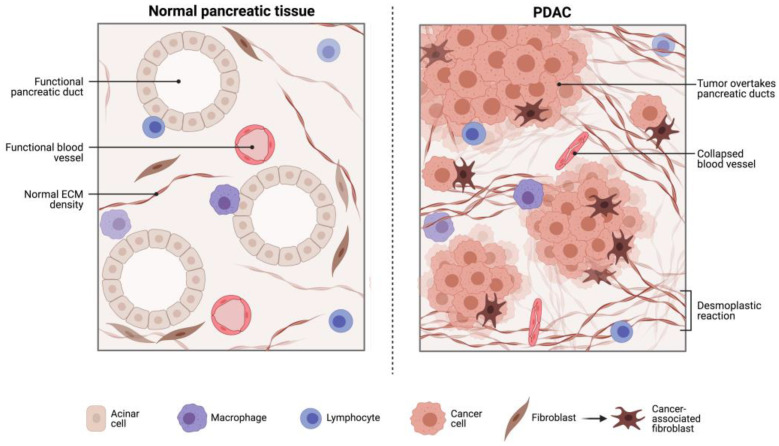
The PDAC TME is heterogenous and poorly characterised. The PDAC TME contains tumour associated macrophages (TAM), myeloid-derived suppressor cells (MDSCs) and regulatory T-cells (T-regs) that are all involved in immunosuppressive tumour promoting activity. Furthermore, there are dense desmoplastic reactions as well as collapsed vessels which all provide barriers to cytotoxic T cell infiltration targeting PDAC tumour cells. The TME affords PDAC protection against chemo- and immune-therapeutics. Reproduced with consent from Merali et al., 2022 [26].

Overall, current findings in the literature are disappointing for PD-1 inhibitor monotherapy in advanced PDAC, as compared to other cancers. The poor response associated with PD-L1 inhibitors in advanced PDAC is likely a reflection of the non-immunogenic nature of PDAC due to the immunosuppressive elements of the TME, heterogeneity associated with this cancer as well as the strong desmoplastic reaction which may act as a barrier to some therapeutic agents [12,14,15,16,17,18,26,27,28] (Figure 1). Moreover, PDAC tumours have many immunosuppressive factors including regulatory T cells (Tregs) and myeloid-derived suppressor cells (MDSCs), making it difficult for immune cells to recognise and eliminate cancer cells [9,10,12,18,28,29,30]. PDAC tumours also have a low mutational burden, which limits the number of neoantigens that can be presented to T cells [9]. This results in a weak or non-existent T cell response.

The only current recommended PD-1 inhibitor for PDAC is Pembrolizumab. It is only recommended for patients with Microsatelitte Instability-High/Deficient Mismatch Repair Molecular subtype (MSI-H/dMMR subtypes), an indicator for efficacy. Unfortauntely only 2% of patients with PDAC have these subtypes [31]. This may in part explain the poor efficacy noted in many studies above.

Because of the apparent ineffectiveness of monotherapy, researchers have sought to investigate the efficacy of combining PD-1 inhibitors with other immunotherapies in order to target PDAC.

#### 2.1.1. Combination PD-1 Inhibitor with Anti-CD25

The combination therapy of PD-1 inhibitors and CD25 (interleukin-2 receptor alpha chain) inhibitors has shown potential synergy in cancer treatment. PD-1 inhibitors block the PD-1 receptor on T cells and CD25 inhibitors target the IL-2 receptor alpha chain, which is expessed on regulatory T cells (Tregs) that suppress immune responses. By inhibiting CD25, the population of Tregs decreases, allowing for a stronger T cell response against tumours. The mechanism underlying the synergy between PD-1 and CD25 inhibitors lies in their complementary effects on the immune system [32]. PD-1 inhibitors remove the inhibitory signals that prevent T cells from attacking cancer cells, while CD25 inhibitors reduce Treg-mediated suppression, which can hinder an effective immune response [32]. CD25 is highly expressed on tumour Treg cells and is a potential target for Treg deletion [32,33]. Vargas et al. found that combining Fc-optimised anti-CD25 treatment and anti-PD-1 antagonists could eradicate solid cancers (advanced kidney cancer and melanoma) by depleting tumour-infiltrating Tregs, which usually downregulate the proliferation of CD8^+^ T cells [34]. Similarly, another pre-clinical trial in a PDAC murine model, found that anti-CD25 combined with anti-PD-1 and TGF-β, inhibited tumour formation and growth, and had a higher cure rate of 80% (n = 5) compared to anti-PD-1 monotherapy (20% cure rate, n = 5) [35]. A reduction in the immunosuppressive Treg counts in the TME was noted in patient’s who received triple therapy over monotherapy. The molecular mechanisms underpinning this was not investigated in the study and warrants further investigations. Limitations to PD-L1 and CD25 include the heterogeneity of tumor microenvironments. PDAC may have an immunosuppressive microenvironment that hampers T cell infiltration and activation, limiting the effectiveness of the combination therapy. The addition of TGF-β to anti-PD-L1 and anti-CD25 does appear to enhance CD8+ TILs infiltration and function in preclinical models [35]. To our knowledge, there are no clinical studies to date exploring the synergistic effect of TGF-β, anti-CD25 and anti-PD-1 therapy in PDAC.

Another potential limitation of such combination therapy is the development of immune-related adverse events due to excessive immune activation. Specifically, such a combination may impact on immune homeostasis as Tregs have a critical role in maintaining immune balance and preventing autoimmune diseases [36]. By targeting CD25 and depleting Tregs, there is a potential risk of inducing autoimmune reactions or exacerbating pre-existing autoimmune conditions [36,37].

#### 2.1.2. Anti-PD-1 and CTLA-4 Inhibitor Combination

PD-1 and CTLA-4 have completely different mechanisms for T cell immune regulation. A phase II clinical study (NCT02558894) compared the effect of combining Tremelimumab (a CTLA-4 inhibitor) and an anti-PD-L1 agent Durvalumab (MEDI4736) with ‘Durvalumab (MEDI4736) monotherapy’ after the failure of 5-FU or Gemcitabine-based chemotherapy, in patients with metastatic PDAC [38]. A response rate of 3.1% (95%CI 0.08–16.22) was observed with combination therapy. Whilst no patients responded to Durvalumab monotherapy. Unfortunately, Durvalumab alone or in combination with Tremelimumab for patients with previously treated metastatic PDAC, was not able to demonstrate any improvement in overall survival (OS). However adverse events were similar between groups who received combination therapy vs monotherapy. The combination of anti-PD-1/anti-PD-L1 and CTLA-4 inhibitors has shown enhanced activity in other tumour types like melanoma and non-small cell lung cancer (NSCLC) [39]. Unfortunately, successes observed in melanoma and NSCLC are not replicable in PDAC. PDAC exhibits a low tumour mutation burden and a limited immune cell infiltration which results in an immunosuppressive microevironment. In addition, some PDAC tumours can express low levels of PD-L1, which makes treatment challenging. There is however a paucity of evidence to support such combination therapies in PDAC at present. An ongoing study (NCT01928394) investigating the efficacy of combining another CTLA-4 inhibitor, Ipilimumab, with Nivolumab (anti-PD-1 agent) in metastatic/advanced solid tumours (including PDAC) is ongoing. However at the time of writing, the results are not yet available. In addition to such limitations of PD-L1 and CTLA-4 inhibitors, adverse reactions include hypothyroidism, colitis, hepatitis, interstitial lung disease, nephritis and encephalitis [40]. Gastrointestinal side effects are the most severe and account for 1–14% of patients. Furthermore, Meserve et al.’s meta-analysis showed a 40% relapse of IBD after administration of PD-L1 or CTLA-4 immune checkpoint inhibitors in 193 patients [41]. The analysis suggested that those treated with CTLA-4 inhibitors may face a higher relapse rate of IBD compared to those who were administered PD-L1.

#### 2.1.3. Combination of Chemotherapy and PD-1 Inhibitor Immunotherapy

Certain chemotherapy agents like gemcitabine and paclitaxel have been reported to have immunomodulatory properties via multiple mechanisms. For example, Gemcitabine upregulates the expression of major histocompatibility complex (MHC) class I and increases the infiltration of T cells and NK cells into the TME to improve the anti-tumour immune response. Studies have also shown Gemcitabine can reverse infiltrating Dendritic cell’s defective cross presentation of tumour antigens [42]. Paclitaxel however has shown to promote the maturation of dendritic cells and increases interleukin-12 (IL-12), which activate T cells and NK cells [43]. Paclitaxel also reduces the number of Tregs in the TME [43]. This makes these agents of great interest. They may expose the immune system to various tumour antigens, reduce immunosuppression, and aid immunotherapeutics. This is the rationale for combining such therapies with PD-1 inhibitors in advanced/metastatic PDAC. In PDAC animal models, a synergistic effect has been observed between the combination of gemcitabine and anti-PD-L1 [44]. This has led to a series of clinical trials investigating the combination of immune checkpoint inhibitor and chemotherapy. A phase Ib/II study (NCT02331251) investigated how Pembrolizumab (PD-1 inhibitor) combined with Gemcitabine and Nab-Paclitaxel chemotherapy reduced PDAC tumour progression rate, in metastatic PDAC (and other tumours) in chemotherapy naïve patients [45]. Progression free survival (PFS) and overall survival (OS) positively correlated (r = 0.777), achieving a median OS and PFS of 15 and 9.1 months respectively in patients with metastatic PDAC. It also showed that such combination therapy can be safely given to chemotherapy naïve patients. That said, patients were highly selected based on their performance status and still suffered a high rate of treatment related adverse events. In fact, the study protocol was amended to include pre-medication with dexamethasone on days of systemic chemotherapy administration to minimise toxicity. Indeed, dexamethasone improved the incidence of certain adverse events and appeared to be well tolerated. Thus, future studies or clinical implementation should consider the addition of dexamethasone to improve treatment associated adverse events associated with such combination therapies. Furthermore, we must consider whether alternative dosing schedules can improve adverse events without compromising other outcomes. A phase I trial (NCT02309177) is underway comparing dosing schedules and combinations of nivolumab (PD-1 inhibitor) with Nab-Paclitaxel with or without gemcitabine for locally advanced/metastatic PDAC. Results are not yet available but eagerly anticipated.

Unlike the other clinical trials which have explored the combination of chemotherapy and PD-1 inhibitors in advanced/metastatic PDAC, one phase II clinical trial (NCT01313416) aimed to evaluate the combination of Gemcitabine and Pidilizumab (CT-011, monoclonal antibody targeting PD-1) in patients who had a primary R0 PDAC resection. Disappointingly, the trial has been discontinued because of drug supply issues and the small sample size of 2 enrolled patients does not provide any meaningful data. Furthermore, comparing any results described in this study with clinical practice is challenging when we consider gemcitabine is not the chemotherapeutic of choice in resected PDAC in a fit and able cohort. Interestingly, CT-011 has been studied in mice which demonstrated reduced tumour growth and prolonged survival in PDAC-induced murine model [46].

However, such combinations may increase risk of toxicity and immune-related adverse events [45].

#### 2.1.4. Combination of Radiotherapy and PD-1 Inhibitor Immunotherapy

Interactions between radiotherapy and PD-1 inhibitors have uncovered new mechanisms that improve the efficacy of radiotherapy. The tolerance of radiotherapy in non-small cell lung cancer (NSCLC) patients is increased through the IL-6/MEK/ERK pathway by upregulation of PD-L1 and downregulation of NKG2D [47]. McCarthy et al. reported a case study on an 83-year-old female with locally advanced PDAC [48]. The patient underwent stereo static body radiotherapy (SBRT) followed by pembrolizumab monotherapy. After 2 cycles of pembrolizumab the tumour size and volume decreased with cystic degeneration. Furthermore, there was no residual dysplasia or carcinoma in the pancreas post-treatment. In addition, Azad et al. demonstrated that inhibiting PD-L1 enhanced the tumours response to high doses of radiation (12 Gy, 5 × 3 Gy, 20 Gy) but not for lower doses of radiation [49]. Greater tumour response was associated with increased numbers and activation of T-cells within the tumour, decreased infiltration of myeloid cells and was dependent on the presence of CD8+ T cells. Additionally, the PD-L1 blockade enhanced the efficacy of radiotherapy in preventing the development of liver metastases.

However, Klug et al. demonstrated a higher level of T cell infiltration when RT5 mice were irradiated with single doses of radiation (0.5 to 6 Gy) [50]. This increase was accompanied by immunosuppressive regulatory T cells CD11b+ and FoxP3+. Interestingly, infiltration of T cells gradually returned to baseline levels as the radiation doses increased to 1, 2 and 6 Gy. Furthermore, there was increased infiltration of transferred tag-specifric TCR transgenic CD8+ or CD4+ cells with irradiated tumours vs unirradiated tumours. The decrease of T cell infiltration at higher radiation levels was thought to be as a result of dose-dependent lymphopenia as a result of irradiation to the spleen. However, infiltration of T cells werewas unaffected in mouse models that underwent splenectomy and/or when tumours were placed in a different location. Therefore, T cell migration may be a result of local effects within the irradiated tumour microenvironment.

#### 2.1.5. Conclusion of Programmed Death Checkpoint Inhibitors

Currently, although numerous clinical trials have shown favourable outcomes for monotherapy with PD-1 immunotherapy in the treatment of various tumours, the evidence underpinning their efficacy in PDAC shows some promise for combination therapies only. Yet the evidence remains limited. Clinical implementation of PD-1 immunotherapies have potential complications and should not be taken lightly in the context of the available evidence. Most cited adverse effects from immunotherapies include rashes, encephalitis, colitis, endocrine diseases, and hepatitis [40,41,51]. Severe and potentially fatal immune-related adverse effects may occur because of T-cell activation and proliferation [52,53]. In addition, Costa et al. reported an extremely rapid pneumonitis within 24 h after use of Nivolumab for PDAC [54]. Furthermore, whilst combination therapies have produced promising results in comparison to mono-immunotherapy, trials suggest that those who receive only immunotherapy have fewer related ≥ grade 3 adverse immune related events (16.5%) compared to immunotherapy and chemotherapy combination therapy (41.09%) [55]. Certainly, if such adverse events are severe, these complications could limit patient compliance and result in delayed/missed doses/incomplete courses of certain therapeutics whether it be the immunotherapy or the chemotherapy. As previously mentioned above, dexamethasone may serve a purpose in chemotherapy and PD-1 inhibitor combination therapy. However, dexamethasone is riddled with potential adverse events/complications itself. In a typically heterogeneous disease state such as PDAC, emphasis should be placed on establishing/investigating biomarkers which could predict therapeutic success as well as risk of serious adverse events and in doing so, facilitate patient tailored therapies to maximise compliance, minimise adverse events and maximise survival outcomes. Unfortunately, in trials to date, there are small sample sizes with only a proportion of patients who respond to therapy, limiting the appraisal of valid biomarkers which correlate with any clinical benefit or adverse event. It is apparent that the use of such therapy in PDAC is limited however some patient groups may confer a benefit. Emphasis should be placed on identifying biomarkers which correlate with a clinical benefit in specific patient groups in order to aid clinical decision making regarding which patients would benefit from such therapies.

### 2.2. CTLA-4 Inhibitors

Cytotoxic T-lymphocyte-associated protein 4 (CTLA-4) inhibitors target PDAC cells. The CTLA-4 protein helps recruit CD4^+^ T-helper cells, which then bind onto antigen presenting cells (APCs) via the CD80 and CD86 receptors.

Unfortunately, except for mismatch repair deficiencies, PDAC tumours largely resist CTLA-4 inhibitor monotherapy. This is because, as previously discussed, the TME is highly specialized to suppress the immune system. A study on PDAC mouse models found no significant increase in CD8^+^ T-cell recruitment with the influx of CD4^+^ cells, unlike previous studies done for metastatic melanoma that showed a large recruitment of CD8^+^ T-cells [56]. This coupled with the unique immunosuppressive TME in PDAC, may in part explain why several clinical phase II trials in PDAC patients using antibodies against CTLA-4 (e.g., Ipilimumab and Tremelimumab) have been unable to confer an impact on OS. For example, one phase II open label study (NCT02527434) evaluated Tremelimumab (a human IgG2 mAb CTLA-4 inhibitor) as monotherapy treatment in patients with metastatic PDAC. This group found that 18 out of 20 patients developed progressive disease and had a poor median OS of 4 months (95% CI 2.83–5.42). Whilst a phase II trial (NCT00112580) investigating the role of Ipilimumab in pre-treated patients with locally advanced or metastatic disease, showed an OS of 4.5 months [57]. Whilst results to date for monotherapy CTLA-4 inhibitor agents are disappointing in PDAC, combination therapies in addition to CTLA-4 inhibitors are showing promise as discussed below.

Specifically, trials have combined GVAX with CTLA-4 in a bid to induce an anti-tumour T cell response. A phase I trial (NCT00836407) comparing ipilimumab and GVAX versus ipilimumab alone as second line therapy for unresectable/metastatic PDAC has shown an OS of 5.7 months with combination therapy compared to 3.6 months with ipilimumab. More recently Hopkins et al. examined specimens from cohorts enrolled in the NCT00836407 and NCT02243371 trials [58]. This group found that patients with PDAC receiving Ipilimumab or Nivolumab with or without GVAX and CRS-207 showed enhanced T-cell responses, as measured by peripheral T cell receptor (TCR) repertoires. Notably, only subjects from the Ipilimumab cohort had prolonged survival. This may be explained by their findings that patients receiving ipilimumab experienced larger changes in their TCR repertoires, especially when GVAC and ipilimumab were used in combination. This group also demonstrated that TCR repertoire analysis can be used as a marker (before treatment initiation and/or after 3 anti-CTLA-4 treatments) in order to identify subpopulations of patients due to be treated with anti-CTLA-4 therapy who will confer a long-term survival benefit (i.e., >6 months). This paves the way for markers which can be used to predict anti-CTLA-4 clinical response. These findings could not be compared with patients treated with Nivolumab (PD-1 inhibitor) as PD-1 inhibitors had a higher clonal repertoire.

Other clinical trials have shed some light on the combination of CTLA-4 inhibition immunotherapy with chemotherapy. For example, a phase I study combined Gemcitabine with Tremelimumab for metastatic PDAC and produced prolonged median overall survival compared with monotherapy using Tremelimumab alone, with an overall survival of 5.3, 8.0 and 7.5 months for patients who received 6, 10 and 15 mg/kg of Tremelimumab respectively. Additionally, seven patients showed stable disease for more than ten weeks and two patients on 15 mg/kg Tremelimumab achieved a partial response at eight weeks [59]. The response rate across this trial was 10.5%. Finally, Kamath et al. conducted a phase Ib study (NCT01473940) combining Ipilimumab with Gemcitabine for 31 patients with advanced or metastatic PDAC disease who have failed conventional treatments [60]. An objective response rate was 14% and the median response duration for the three responders was 11 months, with one patient’s response duration reaching 19.8 months. Median progression-free survival was 2.78 months (95% CI 1.61–4.83 months), and median OS was 6.9 months (95% CI 2.63–9.57 months). Overall, results demonstrated a partial response to PDAC tumours and achieved stable disease. The response rate noted in these trials is comparable to historical trials investigating gemcitabine or FOLFIRINOX alone in advanced PDAC [61,62,63,64]. Furthermore, the median overall survival is less or similar to other trials exploring chemotherapeutics in metastatic PDAC disease [63,64]. This study may suggest that there are some patients who may incur a potential benefit from such therapies over others. The focus of future trials should be on harnessing the potential of multi-centre, collaborative trials to maximise cohort sizes and in doing so identify other patient characteristics and markers which are associated with a therapeutic response.

### 2.3. The Microbiome and Immune Check Point Inhibitors

It is now well established that pancreatic cancer tissues harbor a distinct microbial community that facilitates the acquisition of cancer hallmarks [65]. Distinct changes in the gut and tumour microbiome have been associated with pancreatic cancer progression as well as response to specific therapies [26]. It has previously been shown that commensal gastrointestinal bacteria can influence the efficacy of PD-1 based immunotherapy for various epitheial tumours [66]. In fact, in PDAC murine models, several studies have shown that antimicrobial ablation of the gut microbiome may increase the sensitivity of tumours to immune checkpoint inhibitors and reduce tumour burden [67,68,69]. The use of an antimicrobial cocktail resulted in immunogenic reprogramming of the TME towards an activation phenotype. Findings included increased CD8+ T cell infiltration, Th1 polarisation of CD4+ T cels and M1 macrophage differentiation, with upregulation of PD-1 expression in intra-tumoural effector T cells [67]. This overall suggested alteration of the microbiome may alter immune infiltration and the milieu of immune cells that infiltrate the tumour. Thus providing a favourable TME for PD-1 inhibitors. Facilitating PD-1 blockers working in synergy with antibiotics in this study. The role of combination therapies involving antibiotics and check point inhibitors has not been previously investigated in clinical studies but is worth future consideration. However, the use of broad-spectrum antibiotics in high-risk groups should be considered with caution. Our group have previously written an extensive review of the role of the microbiome in PDAC including its role in immunotherapeutics and how it can be harnessed through microbiome modulation [26].

### 2.4. CAR T-Cell Therapy

CAR (Chimeric Antigen Receptor)-T cell therapy is a form of adoptive cell transfer therapy. CAR-T cells can target any given extracellular molecular structure recognizable by an antibody [12]. Ultimately, it makes tumour cells more recognisable to T cells and may improve the immunosuppressive tumour microenvironment. T cells are collected via leukapheresis, manipulated to target the specific tumour antigen, expanded and then reinfused into the patient [17]. Usually, T-cells are genetically engineered to express CAR cell surface receptors to target the tumour [70,71]. Most research on modified CAR T-cells have investigated how they target mesothelin, a protein found in 80–85% of pancreatic adenocarcinomas. Mesothelin is overexpressed in many pancreatic adenocarcinoma cases and has high abundance on the tumour itself, meaning it has become a prominent target for CAR T-cell therapy [72,73]. Beatty et al. were the first to investigate interactions between CAR T-cell therapy and mesothelin via eight injections of CAR T meso cells intravenously then intratumorally for patients with advanced chemotherapy refractory PDAC [70]. They demonstrated the potential for mRNA engineered T cells to transiently express a specific CAR in order to mediate antitumour activity in advanced PDAC patients, whilst minimising any collateral toxicity. Furthermore, Rojas et al. designed mRNA engingeered T cell vaccinations (cevumeran) by evaluating the DNA and RNA from resected tumours and combined this with atezolizumab and mFOLFORINOX [74]. 50% had T-cell responders to more than one neoantigen and the other half responded to the single neoantigen. In all responders, there was no recurrence 18 months post-operatively. A phase I clinical trial (NCT01897415) conducted by the same group further investigated T-cells transiently expressing anti-mesothelin CAR [71]. Six patients with chemo-refractory metastatic PDAC were recruited and tumour metabolic active volume was measured with a FDG PET/CT scan. The metabolically active volume remained stable for three out of six patients and decreased by 69.2% in one patient, though for this patient only the liver lesions had a complete response, with no effect on the primary tumour. Two of the six patients demonstrated a PFS of 3.8 and 5.4 months. Whilst stable disease was possible, improvement in PDAC survival with CAR-T cell therapy has not been proven in studies to date. In recent ongoing studies (NCT03182803, NCT03030001), CAR T-cells have been genetically-engineered to target mesothelin as well as PD-1 and CTLA-4 antibodies.

Pre-clinical studies have investigated the role of CAR T-cells targeting other receptors such as CEA, HER2, MUC1, CD133 and prostate stem cell antigen (PSCA), in PDAC models [73,75,76,77,78,79,80]. Most interestingly, Chmielewski et al. showed long-term anti-tumour response in orthotopic PDAC murine models administered with CEA directed CAR T-cell therapy, without damage to surrounding normal tissue with lower CEA expression [76]. A phase I trial investigated HER2-targetted CAR T-cell therapy in patients with advanced PDAC and other biliary tract cancers [81]. A median PFS at 4.8 months (95% CI 1.5–8.3 months) was achieved. 5 out of the 11 treated patients achieved stable disease whilst 2 other patients achieved partial response and the remaining 5 patients had progression of disease.

The unique challenge limiting clinical implementation of CAR-T (beyond robust clinical evidence supporting CAR-T cell therapy) is that this therapy may trigger a characteristic toxicity known as cytokine release syndrome (CRS) which can result in pyrexia, hypotension and significant morbidity [82]. This complication is a result of CAR-T cell therapy targeting antigens on normal tissue. However, some studies have shown the safety and feasibility of CAR-T therapy and how to circumvent the potential off-target risk of CRS [71,80]. None the less, the ideal antigen target for CAR-T is one which is exclusively expressed in PDAC cells and unfortunately, all of the antigen targets in the studies detailed above are also expressed in normal tissues [17]. The benefit of targeting a tumour antigen expressed solely in the tissue of tumour origin has been shown for other cancers. We suspect that as our understanding of tumour antigens and the TME continues to grow in PDAC, so will the field of CAR-T therapy and the mitigation of potential toxicity. In fact, in PDAC, one group have recently identified a protein, CEACAM7, which may be exclusively expressed in PDAC cells [83]. Furthermore, they have shown that CAR-T therapy targeting CEACAM7 can result in remission of late-stage patient derived PDAC xenograft tumours in immunodeficient murine models for aggressive metastatic disease. Of course, an immunodeficient state in murine models is not exactly reflective of the PDAC TME. It may be that the role of CAR-T therapy targeting CEACAM7 lies in combination with other immunotherapies targeting the TME in order to make it an immunocompliant environment. This may serve as a potential treatment target in the future for both CAR-T therapy used in isolation or in combination with other novel therapies. Whilst we anticipate both the targeting of this protein and combination therapies in clinical trials in the future, concerns have been raised regarding CAR-T therapy and challenges associated with tumour heterogeneity which may compromise the efficacy of T cell based formulations [29]. Furthermore, the long-term efficacy of CAR-T therapy has been questioned by several studies demonstrating tumour cell driven downregulation or loss of the target tumour antigen over time in other cancers [84,85]. There are many challenges to address in CAR-T therapy in PDAC however the ultimate rate limiting step is our understanding of tumour cell surface target antigens.

### 2.5. KRAS Vaccines

The KRAS mutated proto-oncogene has been found in 90% of PDAC patients, making it a credible target. The KRAS protein controls GTPase, which in turn instructs cancer cells to proliferate and divide via the RAL protein [86,87]. The activation of KRAS triggers downstream proteins such as MEK, RAF, PI3K, AKT and mTOR proteins, to induce transcription, endocytosis, migration and metastasis of cancer cells [88]. KRAS vaccines may provide tumor specific treatments in PDAC. They promote oncogenic KRAS antitumor immunity by providing oncogenic KRAS neoantigens to MHC molecules which aim to develop cancer specific long term memory T cells. Activated T cells attack cancer cells via TC-MHC binding. Certain tumour cells can enter a dormant state following Kras inactivation, remaining quiescent for an extended period. Highlighting the significance of continuous Kras signalling in PDAC development and emphasizes the potential for dormant tumour cells to resurge upon reactivation. Alluding to potential limitations of such vaccine therapies. Several studies have recently reviewed how the KRAS vaccines boost immune defense in the context of PDAC and its potential benefits/limitations [89,90]. Phase I and II studies examined the effects of MEK inhibitors such as Pimasertib, Refamertinib and MSC1936369B in combination with Gemcitabine [91]. Despite their therapeutic benefits, limitations of MEK inhibitors include toxicity such as ocular and skin effects and acquired resistance. Other phase I, II and III studies have combined AKT, mTOR (Selutinib, Everolimus or Temsirolimus), PI3K (Rigosertib) and multi-kinase. Inhibitors (Sorafenib) with and without Gemcitabine but had no significant clinical benefits, though toxicity Increased when these inhibitors were combined with chemotherapy [88]. Other early phase I trials using PI3K, AKT and/or mTOR as monotherapies for PDAC have been disappointing for RAS-mutant cancers [92]. Targeting the upstream K-Ras as opposed to downstream proteins may be beneficial. One phase I/II trial used adjuvant synthetic mutated RAS peptide vaccines in combination with granulocyte-macrophage colony stimulating factor (GM-CSF) in 48 PDAC patients (10 surgically resected and 38 advanced disease) [93]. 25 patients (58%) demonstrated an immunological response to vaccination. Of the 10 who had surgical resections, 1 withdrew due to disease progression. Of the remaining 9, a mean survival was observed of 25.6 months. However, in the study it is unclear if these patients had established adjuvant therapies and thus difficult to ascertain the significance of this survival. In the non-resectable cancer, 32% had stable disease after vaccination and all those with stable disease exhibited an immune response whilst all those who did not exhibit an immune response had disease progression. Thus it is not surprising that generation of a RAS-specific immune response was associated with longer survival with an overall median survival of 148 days in responders vs 61 days in non-responders (*p* < 0002). No patients exhibited signs of toxicity which is encouraging.

### 2.6. Telomerase Vaccines

Telomerase is mainly expressed in cancer cells but not normal cells [94]. Its activation contributes to malignant transformation in pancreatic cells. Telomerase occupies 85–90% of PDAC tumours and is critical in tumour cell immortality [95]. Researchers are now beginning to understand how immunogenic peptides could be used to target cancer cells [9]. The main telomerase-targeting vaccine (GV1001) binds to multiple HLA class molecules to stimulate the release of CD4^+^/CD8^+^ T-cells and target the tumour [96]. In particular, it has been hypothesized that temozolomide may enhance immune response by promoting the release of antigens from tumour cells or modulating the immune tolerance within the tumour microenvironment [97,98]. However, disparities in this vaccine’s effectiveness exist and overall, results are not promising. In a phase I/II clinical study, 48 patients with unresectable PDAC tumours received three different intradermal injections of GV1001 alongside GM-CSF for 10 weeks [93]. Those who received the intermediate doses had a median survival of 8.6 months–significantly higher than that in both the low (4 months median survival, *p* = 0.006) and high-dose (5.1 months median survival, *p* = 0.05) groups. This trial further noted a 25% one-year survival of PDAC patients on the intermediate dose of the vaccine and observed an immune response in 63% of patients, with an OS of 7.2 months versus 2.9 months in non-immune responders. Immunogenic respose was correated with prolonged survival. In contrast, a phase III trial followed up 290 patients with non-resectable PDAC for 6 months (IQR 2.4–12.2) to compare 3 groups; chemotherapy(gem/cap), sequential chemoimmunotherapy with vaccine and gem/cap or concurrent chemoimmunotherapy with vaccine and gem/cap [99]. Median overall survival was not significantly different: chemotherapy was 7.9 months versus sequential chemoimmunotherapy (6.9 months) versus concurrent chemoimmunotherapy (8.4 months). Thus, adding the GV1001 telomerase vaccination did not prolong PDAC patients’ survival and arguably, slightly shortened it when used sequentially. Also, a later phase III randomised trial further compared chemotherapy agents (Gemcitabine and Capecitabine) with and without the telomerase vaccine GV1001 in those with locally advanced or metastatic pancreatic cancer. No difference in overall survival between chemotherapy agents plus the vaccination to chemotherapy agents alone resulted, and the vaccine alone was not statistically different from chemotherapy agents alone (*p* < 0.0175) [91]. This may in part be explained by findings that immune responses to Telomerase vaccines in combination with chemotherapy are often weak and transient [100]. Furthermore the immune mechanism dependends on APCs in the skin and their presentation to epitopes of the vaccine. Overall, studies with larger sample size did not show any current role for GV1001, however the contrasting results from Bernhardt et al. is testament to the fact there may be a role for GV1001 in a patient group we do not yet have the capabilities to understand [101]. It would be of interest to investigate cohort characteristics and markers which may aid defining those who benefit from GV1001.

### 2.7. Gastrin Vaccines

The interaction of gastrin at the CCK (Cholecystokinin) receptor has been found to lead to tumour growth as autocrine, paracrine and endocrine growth pathways cause tumour-associated fibrosis and tumour immune cell alterations [102,103]. The Polyclonal Ab Stimulator (PAS) vaccine has prolonged pancreatic cancer patients’ survival by generating both a humoral and cellular immune response [104]. Studies have demonstrated the importance of both PAS monotherapy for cytokine release upon re-stimulation with gastrin and of PAS and PD-1 antibody combination therapy for T cell-mediated tumour death and memory [105,106]. Gilliam et al. showed how patients who had PAS monotherapy developed neutralising antibodies and that 25% survived 305 days [95]. However, combination therapy (PAS and PD-1 antibodies) produced a median survival of 190 days compared with 84 days for the placebo group. Longer-term survival may be because of less fibrosis, fewer inhibitory Treg lymphocytes and fewer tumour-associated macrophages. Furthermore, longer-term survivors (alive after week 104) seemingly elicited a T-cell memory response besides anti-gastrin neutralising antibodies, perhaps explaining why these patients survive longer than others.

Other pre-clinical studies have compared monotherapy to combination therapy. Osborne et al. reported that PAS-treated C57B/L Kras+/LSL-G12D; Trp53+/LSL-R172H; Pdx-Cre (KPC) mice survived longer, even compared with other mice of the same final tumour weight. KPC mice under the control group survived an average of 50 days, PD-1 antibody mice survived 54 days, PAS monotherapy-treated mice survived 67 days and mice treated with PAS and PD-1 antibody combinations survived 70 days [106,107]. The data suggested not only that PAS vaccines prolong survival but also that it decreased the tumour’s metastatic potential, which may in turn further prolong survival. There may also be a benefit of combination therapies. Furthermore, one study has demonstrated significantly improved survival in 18 patients who received the PAS vaccine compared with placebo patients (median 7.9 months versus 4.5 months; 1-year survival: 33 versus 11%, respectively; log rank *p* = 0.02). Although these results suggest a greater benefit for patients in early-stage disease, another double-blinded, placebo-controlled clinical trial showed a nearly 2-fold increase in median overall survival in chemotherapy-refractory advance-staged PDAC patients taking the PAS vaccine compared with placebo patients [95]. Such vaccines could represent a new therapeutic target for PDAC.

### 2.8. Survivin-Targeting Vaccines

Survivin is required for mitosis, cell proliferation and cell viability [108]. Survivin is highly expressed on pancreatic tumour cells and allows them to avoid apoptosis, making it a credible target for immunotherapy treatments aiming to activate lymphocytes and kill tumour cells with high survivin expression [23,109]. Reassuringly, it’s expression is largely limited to cancerous cells, which may minimise toxicity to normal cells. Inhibition of survivin through siRNA and infection through adenoviruses has increased tumour cell apoptosis, which suggests survivin is crucial for PDAC tumour cell survival and can be targeted. However, whether survivin vaccinations have a role in improving survival in pre-clinical PDAC murine models has brought contention. Some advocate this vaccination, and one small study has shown a survivin based vaccine (plus α-interferon) used in 6 patients with advanced pancreatic cancer (treatment naive and pre-treated) can evoke an immune response with clinical benefit in up to 50% of patients [107]. Ishizaki et al. noted significant differences in survival in C57BL/6 mice when combining MVA-survivin with Gemcitabine versus Gemcitabine alone (*p* < 0.05 by log-rank test) [104]. Similarly, Zhu et al. demonstrated how mice immunised with human survivin had significantly longer lives than those having just the vector control (*p* < 0.05) as well as a greater lymphocyte infiltration [13,105]. Likewise, Shima et al.’s randomised phase II trial found significantly longer post-progression life when combining survivin-2B (SVN-2B) with IFNB (interferon-beta) compared with their placebo (median [95% CI], 312 days (43–460) versus 39 days [9,13,13,14,14,15,16,17,18,19,20,21,22,23,23,24,25,26,27,28,29,30,31,32,33,34,35,36,37,38,39,40,41,42,43,44,45,46,47,48,49,50,51,52,53,54,55,56,57,58,59,60,61,62,63,64,65,66,67,68,69,70,71,72,73,74,75,76,77,78,79,80,81,82,83,84,85,86,87,88,89,90,91,92,93,94,95,95,96,97,98,99,100,101,102,103,104,104,105,105,106,107,108,109,110,111,112,112,113,114,115,116,117,118,119,120,121,122,123,124,125,126,127,128,129,129,130,131,132,133,134,135,136,137,138,139,140,141,142,143,144]; *p* = 0.318) [110]. Also, a case study revealed complete remission (for 8 months) of a 72-year-old man’s with Gemcitabine-refractory PDAC following a modified HLA-A2+-restricted survivin vaccine [111]. However, when he was weaned from the vaccination, he developed recurrent disease. In contrast, Kubo et al. also found that the survivin-based vaccination did not prolong survival, but they noted large infiltrations of CD8^+^ and PD-1 lymphocytes in patients who had received the vaccine, which eludes to its therapeutic potential [112,113]. Despite the current evidence suggesting a promising role for survivin based vaccine, there are no current active clinical trials exploring its role in PDAC. Ongoing evaluation of Survivin as as a therapeutic target with other immunotherapeutics may have future potential.

### 2.9. Heat-Shock Protein (HSP) Peptide Complex-Based Vaccines

The HSP-peptide complex-based vaccines were the first anti-cancer vaccines derived from resected tumour tissues and have been shown to induce a tumour immune response [112,114,115,116]. They suppress tumour growth via two mechanisms: firstly they may cause heat shock or oxidative stress, they may induce the expression of HSP110 and GRP170, which aids the restoration of protein folding and cellular function to counter proteotoxic stresses and thus promote cell survival; and secondly, HSP-peptide complexes presented on MHC class I and II molecules activate CD4^+^/CD8^+^ T-lymphocytes [112,117]. Whether these heat-shock proteins lead to both innate and adaptive immune responses remain unknown. Nevertheless, studies have demonstrated anti-tumour immune responses. For example, in a phase I trial, 10 PDAC patients with resected stages I/II without adjuvant chemoradiation were vaccinated with HSP96-peptide complexes weekly (four times) and demonstrated a median overall survival of 2.2 years [118]. None of these patients received any form of adjuvant therapy and the treatment was well tolerated. Although HSP-peptide complex-based vaccines appear promising for pancreatic cancer patients, Maki et al. have demonstrated it can be challenging to yield enough HSP peptide complex-based vaccine preparations from resected tumours specimens [118].

### 2.10. MUC-1 Targeting Vaccines

PDAC patients have an overexpression of MUC-1 proteins on tumour cells, indicating another credible target for treatment [119]. Kondo et al. demonstrated how combining cytotoxic T-lymphocytes (CTLs) and Mucin 1 cell surface associated dendritic cells (MUC-1-DCs) resulted in 5 out of 20 patients having stable disease after unresectable, recurrent PDAC, giving an overall survival rate of 9.8 months [120]. Shindo et al. compared MUC1-DCs versus combined MUC1-CTLs and Gemcitabine in 42 patients with either unresectable or recurrent PDAC. Patients who received the combination therapy had a median survival of 13.9 months and a disease control rate of 60% [121]. Demonstrating it may inhibit tumour growth and prolong survival. This clearly indicates the potential clinical application of MUC-1 as a tumour associated antigen. There are unfortunately no MUC-1 targeting vaccine phase III clinical trials yet. The stark differene in improved survival between these studies may in part due to sample size as well as patient characteristics such as chemotherapy combinations, frequency of distant metastasis in cohorts as well as MUC1 mRNA transfection rates. Another study demonstrated that CAR T-cells engineered to recognise the tumour-specific Tn glycoform of MUC1 neoantigen controlled tumour growth and prolonged survival [80]. Another phase I study investigated combined CEA and MUC1 vaccines in ten patients, resulting in a 6.3-months overall survival time [122]. In this study, patients who developed a CEA and/or MUC-1 specific immune response had a significantly increased OS (15.1 months vs 3.9 months, *p* = 0.002). However, a larger phase III randomised clinical trial did not advocate this vaccine’s use after comparing another form of MUC1 vaccine (PANVAC-V) to a Gemcitabine-based chemotherapy regimen, as the former did not improve overall survival compared with the latter [123]. Results for studies investigating the MUC1 targeting therapies have shown little efficacy to date with the most promising results demonstrated when used in combination with chemotherapy. CAR-T therapy targeting MUC-1 positive solid tumours (including PDAC patients who have had an R0 resection) is currently being investigated in a phase I/II clinical study (NCT02587689). This trial is in the recruitment phase. There are no other trials ongoing in this specific field.

### 2.11. Listeria-Based Vaccines

Listeria-based vaccines have been used to express mesothelin, which is highly expressed in PDAC tumours. Once the vaccine is bound to mesothelin, CD8^+^ T-cells get released, resulting in tumour lysis. One phase I open label, multiple-dose, dose escalation (NCT00585845) study investigated the effects of a dose escalation of a listeria vaccine (ANZ-100) alongside a live attenuation of the listeria vaccine expressing mesothelin in 7 patients with PDAC. Only 3 of the 7 PDAC patients survived more than 15 months and interestingly all 3 had received prior GM-CSF vaccine therapy [124]. The administration remained safe and resulted in immune activation. Unfortunately, this was a small cohort, the trial failed to meet its primary end point which was OS improvement and durable response rates were not registered. Furthermore, in addition to PDAC, cohorts included patients with ovarian, mesothelioma and lung cancer. Combination studies involving the Listeria vaccine have not been promising to date. Including a phase II study (NCT02243371) comparing combined GVAX and Cyclophosphamide alone and with CRS-207 (an attenuated strain of *Listeria monocytogenes* engineered to express mesothelin) for 90 patients with metastatic PDAC [125]. This trial demonstrated an improved objective response rate but failed to demonstrate overall survival benefit. A larger phase IIb study of 303 PDAC patients compared combined GVAX, Cyclophosphamide and CRS-207 versus CRS-207 alone and versus chemotherapy alone, respectively giving survival times of 3.8 months, 5.4 months and 4.6 months [126]. Unfortunately, this trial did not meet its primary efficacy endpoints and did not demonstrate a survival benefit over chemotherapy. However, OS amongst the CRS-207 monotherapy group was better than chemotherapy. Whilst this was minimal (5.4 versus 4.6), it may be meaningful in a treatment refractory cohort. These results should be further contextualized with an understanding that the CRS-207 alone treatment group had a significantly higher median baseline Ca-199 (1114.9 versus 411 in GVAX+Cyc+CRS-207 group) versus 150 in the chemotherapy alone group). It should further be noted that there was a high dropout rate in the chemotherapy arm of the trial following randomized. This is of course the difficulty associated with running open label RCTs. The study hypothesized that patients had likely failed previous course of chemotherapy and withdrew after randomization to further chemotherapy in a bid to pursue novel agents. Treatment related adverse events were reported in 46.8%, 36.8% and 27.8% in the three groups respectively. Suggesting combination therapies have a greater rate of AE and the vaccine monotherapy had more AE compared to chemotherapy alone. Whilst no treatment related deaths were noted, these findings highlight that implementation of such vaccine therapies and their associated AE, may have implications on patient compliance, acceptability, and quality of life. This is of considerable significance when we consider that numerically speaking, CRS-207 alone had a marginally longer survival time. However one must question the benefit of this if a degree of quality of life is not maintained during that time. As previously discussed, immune checkpoint inhibitors used as single agents are ineffective in PDAC. It is postulated that vaccine therapies may sensitize PDAC to immune checkpoint inhibitors by re-programming/priming the immunosuppressive and poorly immunogenic TME for immune checkpoint inhibitors, however this is largely unproven in humans but preclinical studies are promising. Kim et al. also synthesized the ANXA2 coding sequence using Listeria codon bias to demonstrate how Lm-ANXA2 targeting *Listeria* vaccines prolong survival in a transplant model of mouse PDACs [127]. Furthermore, their group showed that the sequential treatment of PDAC murine models with ANXA2-expressing Listeria based immunotherapy followed by anti-pd-1 therapy increased specific T cell responses within the TME.

The attenuated Listeria monocytogenes vaccine has been used to deliver an immunogenic antigen tetanus toxoid protein (TT_856–1313_) to PDAC tumour cells in a murine PDAC model [128]. It has shown promise in activating an antitumour immune response in the TME in order to kill infected tumour cells in vitro as well as in vivo. Listeria-TT_856–1313_ used in combination with low dose gemcitabine in advanced PDAC KrasG12D,p53R172H,Pdx1-Cre (KPC) murine models reduced tumour growth, tumour metabolic activity and tumour burden by 80%, metastasis by 87% and increased survival by 40% compared to non-treated mice. Furthermore, this study compared Listeria carrying Survivin and TT_856–1313_. They found TT_856–1313_ was more immunogenic resulting in a greater T cell response compared to Survivin. Such a combination strategies warrants investigation in humans. Clinical trials are still ongoing investigating this vaccination approach with various combination immunotherapies as well as stereotactic body irradiation (NCT03190265) (NCT03006302) (NCT03153410) (NCT03161379).

### 2.12. Dendritic Cell-Based Vaccines

Dendritic cells (DCs) work by early-recognition of antigens on the tumour surface and prime naïve T-cells to produce memory T-cells and B-cells [129,130]. It has been shown by higher DC levels in serum and tumour analysis correlate with improved survival [131,132]. Gemcitabine seems to enhance DC maturation and enhances the efficacy of DC based vaccines [133,134]. This early recognition of such tumours combined with gemcitabine chemotherapy treatment provides promising results for patients with pancreatic cancer. Several studies have advocated dendritic cell-based vaccines. For example, Kimura et al.’s clinical study revealed a prolonged survival of 360 days from the first vaccination when 49 patients with inoperable pancreatic cancer were immunised with the DC-based vaccine (primed with Wilms Tumour gene protein, which is heavily expressed in PDAC) and treated with Gemcitabine and S-1 [135]. There were a subset of patients with decreased Tregs in peripheral blood which correlated with prolonged OS. From these promising results, another study involved administering 255 patients with inoperable pancreatic cancer a combination of Gemcitabine and the DC-based vaccine [129]. The median overall survival was 16.5 months from diagnosis and 9.9 months from the first vaccination. A prolonged median survival time in patients with positive delayed type hypersensitivity skin reaction emerged compared to that of the patients with a negative reaction (*p* = 0.0157). With these encouraging results, two small-scaled prospective clinical studies were performed for patients with advanced PDAC. Gemcitabine and DC vaccine were combined with a HLA class-I-restricted WT1 peptide for 10 PDAC patients. Three who had DTH positivity had disease control [136]. A further study combined HLA class II-restricted epitope (WT1-II) and HLA class I-restricted epitope (WT1-I) to the dendritic vaccines for ten patients with stage IV PDAC. The survival of seven out of ten patients was significantly prolonged (*p* = 0.036 in OS, *p* = 0.010 in PFS) [137]. Conflicting results have however been reported. Nakamura et al. vaccinated 11 patients who were administered chemotherapy and six patients who were not on any chemotherapy. The DC vaccination resulted in a median survival of 9 months in both groups and no significant difference between combination therapy vs vaccination treatment alone [5]. Of course this study did have a small sample size compared to other studies. Several other promising studies have explored WT-11 generated DC vaccines in PDAC [138,139]. A double-blind RCT evaluating the safety and efficacy of DC vaccine loaded with WT1 peptides in combination with S-1 is planned in patients with advanced PDAC [140].

Other work, such as a Phase I study, has investigated the effect of allogeneic tumour lysate loaded autologous monocyte derived DCs in the treatment of resected PDAC. The vaccination therapy indicated a feasible and safe immune reactivity induction capability [141]. These patients had resected PDAC who had no radiologic signs of recurrence after standard treatment. Seven out of ten patients did not experience disease recurrence or progression at a median follow-up of 25 months (15–32 months).

The main disadvantage of DC based vaccines is the laborious process required to produce this vaccine [142]. Furthermore, DC based vaccines are generally more efficious in early stages of disease due to the tumour progression and immune dysfunction associated with advanced disease [143]. Highlighting the importance of combination therapies which can unlock the potential of DC in advanced disease states.

### 2.13. Oncolytic Viral Therapy

Oncolytic viral therapy (OVT) has shown promise as a potential treatment for pancreatic cancer [144]. It utilises viruses to selectively kill cancer cells via direct lysis or immune-mediated mechanisms e.g., cytokines or co-stimulatory molecules to enhance anti-tumour immune response, while sparing normal cells [145]. One advantage of such mechanisms is the ability to target cancer cells in hard-to-reach locations such as dense stroma of pancreatic tumours [145,146]. OVT is designed to target molecular markers which are overexpressed or dysregulated in cancer cells, in particular KRAS which is present in 90% of pancreatic cancer cases. OVT can also be combined with checkpoint inhibitors to enhance the anti-tumour immune response. For example, Mahalingam et al. evaluated the combination of pembrolizumab with the oncolytic virus pelareorep and chemotherapy (either 5FU, gemcitabine or Irinotecan) and demonstrated that 65% of patients achieved disease control and a median overall survival of 10.5 months [147]. The clinical benefit was demonstrated to be associated with new T cell clones recircuating systemically as well as transcriptional evidence of systemic immune activation. Another RCT has compared pelareorep with or without chemotherapy (carboplatin/paclitaxel) [148]. Median PFS did not differ between cohorts (4.9 vs. 5.2 months respectively, *p* = 0.06). However an increase in pro-inflammatory cytokine like Th1 CD4+ and CD8+ T cells, as well as Tregs, was noted. The conflict in findings might suggest that the initial priming and mobilization of T cells facilitates subsequent immune checkpoint blockade, confers a clinical benefit.

Other work has shown the potential for alternative combination therapies used with chemotherapy and OVT and how one can circumvent potential adverse events associated with systemic administration of OVT. For example, a phase I clinical trial by Hirooka et al. (2018) investigated the safety and efficacy of EUS-guided intratumoral injection of the oncolytic virus HF10 (with erlotinib) in 12 patients with unresectable locally advanced pancreatic cancer [149]. HF10 was well tolerated and showed potential therapeutic effects with 1 out of 12 patients achieving a partial response and 5 out of 12 patients achieving stable disease. The median PFS was 6.3 months and median OS was 15.5 months. Whilst EUS and intra-tumour injection is logical in terms of minimizing systemic effects of treatment, one patient did develop a duodenal perforation following EUS. Thus EUS should be considred with caution. Finally, Singh et al., developed a microRNA-sensitive oncolytic measles virus for chemovirotherapy of pancreatic cancer. This virus was also able to selectively replicate and kill cancer cells while sparing normal cells, leading to tumour regression in mouse models [150].

OVT therapies have a potential to become an effective treatment for PDAC, probably as a combination therapy with other immunotherapeutics/chemotherapeutics. Several trials are exploring this further (NCT02705196; NCT04637698; NCT03252808; NCT02045589; NCT02653313).

### 2.14. Immunomodulators of the TME

The immune microenvironment for PDAC patients is complex. Among the complex and diverse cellular components of the TME, tumour associated macrophages (TAMs) appear early in tumourgenesis and represent the predominant stroma cel fractions that elicit T cell exhaustion and eventually overwhelm anti-tumour cellular immunity in advanced lesions [151,152,153]. Furthermore, TAMs contribute to tumour progression, angiogenesis, anti-inflammation, extracellular matrix remodeling and treatment resistance to gemcitabine [154,155,156]. Three primary strategies have been identified to target tumour associated macrophages (TAMs) effectively: macrophage elimination, recruitment inhibition and reprogramming [157]. A notable player in recent years has been BLZ945, a clinically relevant CSF1R inhibitor. Studies have demonstrated that BLZ945 effectively prevents infiltration of TAMs and promotes their reprogramming to an antitumour phenotype [158,159]. In particular, BLZ945 reshaped the immune landscape, inducing an antitumour cross-talk among TAMs, IFNy-producing NK and T cells, and IL-12 producing dendritic cells [159]. Furthermore, when combined with radiotherapy in preclinical trials, BLZ945 resulted in reprogrammed macrophages, enhanced therapeutic sensitivity, and prevented recurrence in glioma models [160,161]. Further avenues are currently being explored including nanoparticle-based therapies and recombinant protein treatments that may reprogram TAMs to an immune-supportive phenotype [162,163]. Treatment strategies targeting TAMs in PDAC requires an in depth knowledge of macrophage compartments over the course of the disease, which has historically been poorly characterized in PDAC. However recent work has further characterized the macrophage compartment in PDAC which may pave the way for macrophage targeted personalized cancer therapies [164].

The immunosuppressive effects of IDO-Kynurenine pathway have been suggested to contribute to immune escape and promote tumour growth and metastasis in PDAC [165]. Increased levels of kynurenine (a product of tryptophan metabolism) can inhibit the proliferation of NK cells and T cells. Kynurenine has shown to induce apoptosis of specific immune cell subsets and can further modulate the balance of TH1/TH2 cells. Furthermore, the interaction between hypoxia and IDO-Kynurenine pathway promotes tumour aggressiveness. Targeting the IDO-Kynurenine pathway in PDAC may hold promise for immunotherapy and improving treatment outcomes [165]. Epacadostat is the most investigated IDO1 inhibitor in cancer. However, in PDAC there is only one study investigating Epacodostat which is currently active (NCT03006302). This study is a Phase 2 interventional study in patients with metastatic PDAC who had progressed on chemotherapy. Patients will receive epacadostat/pembrolizumab/CRS-207, with or without Cyclophosphamide and GVAX vaccine. Results eagerly anticipated.

Another immunomodulator in PDAC is the anti-CD40 agonist antibody. CD40 is a cell surface member of the TNF receptor family. When activated, it promotes dendritic cell priming of T cells and macrophages. In one phase Ib study, Sotigalimab (APX005M, CD40 agonist), was used in combination with chemotherapy and nivolumab for metastatic PDAC [166]. Among 24 patients, the ORR was 58% and median PFS was 11.7 months. This compares favourably with historical median PFS for chemotherapy alone [64]. The same group are further evaluating these results in a phase II trial and if confirmed in later trials, may prove invaluable in the management of these patients and may even replace chemotherapy alone as the standard of care in such groups. Another phase I study has shown APX005M given neoadjuvantly in resectable PDAC shows a significant increase in T cell enriched tumours upon resection compared to cancers treated with chemoradiation alone or untreated tumours [167]. Larger studies are required to confirm such findings. Other pre-clinical work has shown the potency of DC therapy when used in combination with CD40 agonist FGK45 in PDAC murine modles, and hints towards future scope for clinical trials [168].

## 3. Discussion

Despite recent advances in our understanding of pancreatic cancer, the current overall prognosis is poor [1,2,3,4,7,169]. In various solid cancers, immunotherapy has completely changed the management algorithm [15,170]. Yet this has not been the case for PDAC, despite it being an immunologically cold tumour with a low mutational burden [28]. Which should suggest there is scope for the application of immunotherapeutic strategies. The limited success with immunotherapy in PDAC may be largely due to immunosuppression, dense desmoplastic reaction and immune escape associated with the TME [12,14,15,16,17,18,26,27,28]. Furthermore, PDAC often presents late with metastasis. This may create additional immune barriers [27].

This field of active study remains in its infancy with much yet to be understood and determined on a molecular and clinical level. For example, immunotherapeutic strategies have shown efficacy in subpopulations of study participants in several studies [21,58,81,93,111,120,124,149]. However our understanding of why subpopulations respond is limited. The tumour microenvironment is a heterogenous dynamic environment which dictates tumour behaviour, and it may be that the TME in subpopulations which benefit from treatment is distinctly different to those which do not confer a benefit [171,172,173]. Furthermore, it has been suggested that the presence of metastasis in PDAC, may mask drug activity [27].

**Figure 2:**
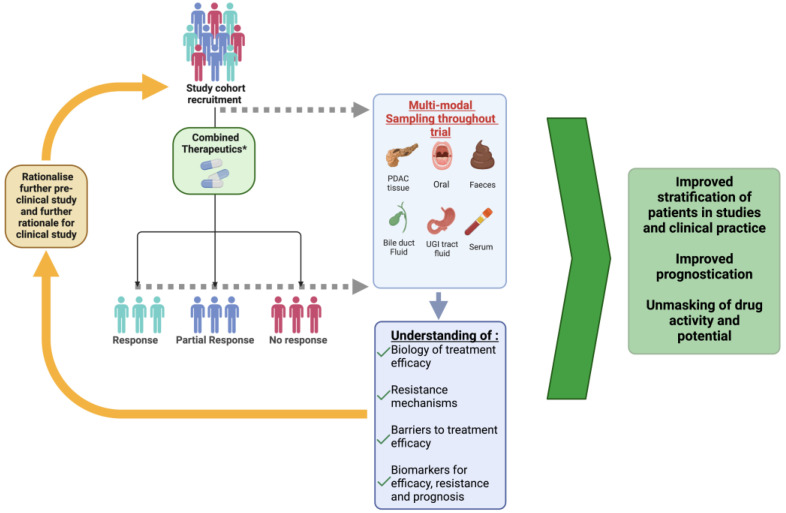
Demonstrating how multi-center study into combination immunotherapeutics can advance therapeutic strategies. Patients are recruited with disease specific characteristics. Prior to initiation of therapy, patients should have multi-modal baseline characteristic sampling carried out from various compartments including but not limited to the above. Dependent on resources, suitability and feasibility of carrying out any given investigation. Sampling should be carried out at defined time points during treatment. Following therapy and identification of response, samples are analyzed. Pharmacodynamics, molecular, immune and microbiome analysis of samples can further our understanding of the biology associated with treatment efficacy, barriers to treatment efficacy and mechanisms associated with resistance or compliance. Furthermore, biomarkers can be appraised for efficacy, resistance, and prognosis. Findings from studies can be used to Rationalize further pre-clinical or clinical study. Ultimately improve the stratification of patients, improve prognostication and unmask drug activity and potential. * Combined therapeutics may include immunotherapy, chemotherapy and/or novel therapeutic adjuncts.

Unfortunately, there has been limited appraisal of biomarkers as surrogates for the tumour immune profile/TME characteristics, many of the studies described above. This may in part be due to the difficulty in obtaining adequate tissue samples to assess biomarkers/carry out immunohistochemistry in patients who have not had a resection [174]. None the less, the value of biomarkers for prognostic and predicting response in chemotherapy [175,176] and immunotherapy [20,21,58,93,121,124,176,177,178,179,180,181] for PDAC has previously been demonstrated in several studies. To investigate and understand biomarkers which help distinguish which patients benefit from such therapies, there should be a continued strive in the community to develop large multi-centre studies. In doing so we may tailor therapies to those who would benefit most from it (Figure 2). In terms of achieving adequate tissue sampling, some studies have been able to circumvent the need for adequate tissue sampling. For example, analysing peripheral TCR repertoires can be used to identify responders to anti-CTLA-4 therapy in PDAC cohorts [58]. Whilst another study has shown prolonged median survival time in patients with positive delayed type hypersensitivity skin reaction emerged compared to that of the patients with a negative reaction (*p* = 0.0157) following immunotherapy [135]. Other studies have also shown the prognostic value of biomarkers in immunotherapy treated PDAC cohorts [180]. Furthermore, it has previously been hypothesized that microbiome constituents such as intratumoral Gammaproteobacteria may have an association with chemoresistance to Gemcitabine [26]. Given the intimate relationship between immune landscape and PDAC intratumoral microbiome, it would be of great interest to explore the role of microbiome biomarkers and efficacy (or lack off) of immunotherapeutics in trials [67]. Less invasive assays for microbiome biomarkers (used in PDAC) include stool and oral microbiome assays [26].

We must continue to strive to understand the TME, proteins expressed exclusive by PDAC cells or indeed tumour antigens expressed solely on PDAC cells. Furthermore, with genomic sequencing, we may be able to identify the most suitable regimens for patients. Selective effective therapeutic strategies in PDAC continue to be a major challenge in the field, however as our understanding increases, so will the credible targets for therapies and the sophistication of future therapies and their success. For example, a recent study has developed a personalized mRNA vaccine (expressing up to 20 neoantigens) that induces neoantigen specific T cells [74]. Neoantigens can be identified by individual tumour analysis, providing patient specific neoantigens for a personalized vaccine. In the Phase I clinical trial, 16 PDAC patients were injected with a personalized mRNA vaccine after surgical resection, in combination with mFOLFIRINOX and atezolizumab. After 18 months, 50% remained cancer free. All these patients had predominant activation of neoantigen-specific CD8 T cells. Such work alludes to the sophistication of treatments and the individualized patient specific approach that may be required in PDAC. This study has been further contextualized in a recent article [182].

Novel immunotherapy agents in PDAC could have a potential to improve overall disease free and survival rates in the future. Therapies targeting immune checkpoints (eg. PD-1 and CTLA-4) have had minimal clinical success in PDAC likely due to the immunosuppressive microenvironment dominated by extracellular matrix proteins, fibroblast subtypes and other immune cell types [183]. There is limited evidence for the role of immunotherapy monotherapy, largely due to cancer development and progression in PDAC. Which means that it is unlikely that any given agent can achieve sufficient control independently However, the combination of two or more immunotherapeutic agents seems promising and may be the key to simultaneously targeting multiple pathways involved in proliferation, survival and spread of PDAC cells [34,60,95,104,105,110,121,124,126]. Therefore, a sound understanding of the underlying immune mechanisms in this heterogenous disease state coupled with greater knowledge of actions of each agent (when coupled/acting in synergy) in the TME as well as the mechanisms which lead to tumour escape, will provide us with multiple potential anti-neoplastic treatment targets and therapeutic options.

There are some potential unique logistical and clinical challenges which will be associated with immunotherapy in PDAC. Conventional therapies are associated with fatigue, daily hospital visits, increases in hospital bed occupancy and increased financial pressures for patient and government [184,185]. Immunotherapies will likely require multimodal treatment approaches to unlock their potential. Thus, if immunotherapies (used in combination with each other or chemotherapeutics) develop an established role in PDAC, it could possibly result in negative implications on cost containment, hospital visits and bed pressures.

This will be compounded by another unique challenge of multimodal therapy; associated toxicity. Several studies have showcased the adverse effects associated with combination therapies [34,35,40,41,45,55,126]. None the less, severe adverse events have only been reported in a minority of patients in studies [40,41,52,53,54,124,126] Several studies have already attempted to circumvent the adverse events associated with immunotherapies with promising results whether its pharmacological interventions or consideration of local instead of systemic administration of immunotherapeutics [45,149]. Future work should continue to improve the safety profile of these systemic therapies and once we have established therapies with positive outcomes, we must consider route, timing and dose adjustment comparisons in order to inimize toxicity whist ensuring optimal drug delivery.

This review is limited by the novelty of this topic which reduces the amount of manuscripts that can be reviewed. Potentially, once more studies emerge with larger sample sizes and set parameters, then meta-analyses on registered clinical trials to compare effect sizes of treatments will become feasible. Many studies reviewed herein were in pre-clinical models, limiting conclusions regarding their clinical application. It has also been noted that some clinical trials derived from published manuscripts may have been outdated by publication date. Another consideration is that positive trials are twice as likely to be published than negative ones further tempting publication bias [186].

## 4. Conclusions

Pancreatic ductal adenocarcinoma (PDAC) often presents with metastasis, which means that the cancer is unresectable at the time of diagnosis. Currently, surgery provides the only curative option for the eligible minority. Conventional treatments such as chemotherapy do prolong survival. However the disease remains associated with a poor 5 year survival. Monotherapy immunotherapy has been largely disappointing in PDAC. This is largely due to the complex, heterogenous TME. Evidence suggests combination therapies show promise. Yet we have not unlocked the potential in PDAC compared to other solid tumours. This is likely, in part, a result of our incomplete knowledge of the underlying disease mechanisms and the intricate relationships between immunotherapy/chemotherapy and the TME. However, this field remains in its infancy in PDAC with much left to uncover. We must strive to understand more on a clinical, immune, and molecular level. Furthermore, exploring the predictive and prognostic biomarkers of this disease are crucial. This knowledge may facilitate both patient stratification and therapies targeting multiple pathways appropriately. Until we have a sound grasp of this knowledge, precision therapies like immunotherapy in PDAC and proving their clinical application will be limited by the knowledge gap. Nonetheless, research to date and academic persistence have begun to pave the way for a future where PDAC may be viewed as a manageable disease state.

## Data Availability

The data presented in this study are available in this article.
